# Age-dependent benefit of neoadjuvant treatment in adenocarcinoma of the esophagus and gastroesophageal junction: a multicenter retrospective observational study of young versus old patients

**DOI:** 10.1097/JS9.0000000000000713

**Published:** 2023-09-14

**Authors:** Ingmar F. Rompen, Nerma Crnovrsanin, Henrik Nienhüser, Kerstin Neuschütz, Lana Fourie, Leila Sisic, Beat P. Müller-Stich, Adrian T. Billeter

**Affiliations:** a Department of General, Visceral and Transplantation Surgery, Heidelberg University Hospital, Heidelberg, Germany; b Netherlands Cancer Institute, Amsterdam, The Netherlands; c Department of Visceral Surgery, Clarunis-University Center for Gastrointestinal and Liver Diseases, St. Clara Hospital and University Hospital Basel, Basel, Switzerland

**Keywords:** esophageal adenocarcinoma, gastroesophageal junction cancer, individualized treatment, neoadjuvant treatment, survival

## Abstract

**Objectives:**

The objective was to provide evidence for age-dependent use of neoadjuvant treatment by clinical comparisons of young (lower quartile, <56.6 years) versus old (upper quartile, >71.3 years) patients with esophageal and esophagogastric-junction adenocarcinoma.

**Background:**

Neoadjuvant treatment is the standard of care for locally advanced and node-positive EAC. However, the effect of age on oncological outcomes is disputable as they are underrepresented in treatment defining randomized controlled trials.

**Methods:**

Patients with EAC undergoing esophagectomy between 2001 and 2022 were retrospectively analyzed from three centers. Patients having distant metastases or clinical UICC-stage I were excluded. Cox proportional hazards regression was used to identify the variables associated with survival benefit.

**Results:**

Neoadjuvant treatment was administered to 185/248 (74.2%) young and 151 out of 248 (60.9%) elderly patients (*P*=0.001). Young age was associated with a significant overall survival (OS) benefit (median OS: 85.6 vs. 29.9 months, hazard ratio 0.62, 95% CI: 0.42–0.92) after neoadjuvant treatment versus surgery alone. In contrast, elderly patients did only experience a survival benefit equaling the length of neoadjuvant treatment itself (median OS: neoadjuvant 32.8 vs. surgery alone 29.3 months, hazard ratio 0.89, 95% CI: 0.63–1.27). Despite the clear difference in median OS benefit, histopathological regression was similar ((Mandard-TRG-1/2: young 30.7 vs. old 36.4%, P= 0.286). More elderly patients had a dose reduction or termination of neoadjuvant treatment (12.4 vs. 40.4%, *P*<0.001).

**Conclusion:**

Old patients benefit less from neoadjuvant treatment compared to younger patients in terms of gain in OS. Since they also experience more side effects requiring dose reduction, upfront surgery should be considered as the primary treatment option in elderly patients.

## Graphical abstract

**Figure:**
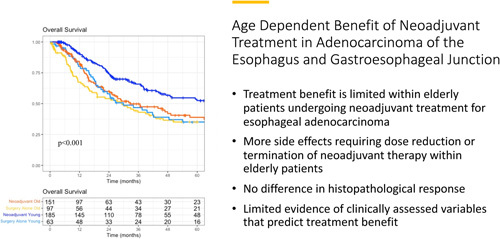

## Introduction

Highlights

- Treatment benefit is limited within elderly patients undergoing neoadjuvant treatment for esophageal adenocarcinoma.
- More side effects requiring dose reduction or termination of neoadjuvant therapy within elderly patients.
- There is no difference in histopathological response.
- There is limited evidence of clinically assessed variables that predict treatment benefit.

Adenocarcinoma of the esophagus and gastroesophageal junction (EAC) as one of the most aggressive types of cancer is related to poor survival^1^. Diagnosis is often made when tumor size is advanced and lymph node involvement is present^2^. In those patients, the introduction of neoadjuvant treatment with FLOT or CROSS has led to an improved overall survival (OS)^3,4^. Current treatment guidelines recommend neoadjuvant treatment before undergoing surgery for all resectable tumors except UICC-stage I disease^5–7^. These guidelines are solely based on the clinical assessment of TNM stage and do not consider other variables such as age or performance status. This lack of individualized treatment may result in the omission of important treatment defining aspects. A major histopathologic response rate of only 37% and recurrence in approximately one out of two resected EAC patients after neoadjuvant treatment moreover indicates that critical selection should be implemented into clinical practice^8–12^.

The role of age in treatment plans for EAC and the effectiveness of neoadjuvant therapy in elderly patients has been intensively discussed because treatment defining randomized controlled trials had a massive underrepresentation of elderly patients in their cohorts^4,13^. The median onset of esophageal adenocarcinoma is 69.6 years in a western population whereas the median ages of the CROSS, MAGIC, and FLOT trials are relevantly younger with 60, 62, and 62 years, respectively^3,4,14^. Only 20% of patients were above 70 years in the MAGIC trial whereas the CROSS trial did exclude patients greater than 75 years of age^4,14^. Furthermore, patients with poor performance status (ECOG ≤2, medical operability), and patients with frequently encountered preconditions in elderly patients that could hamper outcomes (creatinine-clearance <50 ml/min, ejection fraction <50%, NYHA III-IV, active coronary heart disease, severe internal accompanying disease, peripheral neuropathy, hypersensitivity to any used agents) were excluded from the aforementioned treatment defining randomized controlled trials^3^. These strict exclusion criteria lead to poor evidence for the use of neoadjuvant treatment in elderly EAC patients. Given the poor tolerability to these agents, better evidence is much needed^15,16^. On the other hand, early-onset gastrointestinal cancer is often associated with more advanced tumor stages and more aggressive tumor characteristics^17,18^. These surrogate markers hamper prognosis in young patients with gastrointestinal cancers but indicate a potential benefit from aggressive cytotoxic treatment^17,18^.

The aim of this analysis was to provide evidence for age-dependent use of neoadjuvant treatment by clinical comparisons of young and old patients with EAC.

## Methods

### Study design

This analysis represents a retrospective, multicenter cohort study of surgically treated patients with EAC with or without preceding neoadjuvant treatment. Data were gathered from medical records and patient interviews. The study was performed in compliance with the STROBE and STROCSS guidelines^19,20^, Supplemental Digital Content 2, http://links.lww.com/JS9/A975. The study was approved by the Ethics Committee of Heidelberg University (S-649-2012) and complied with the 1964 Helsinki Declaration and its later amendments. All included patients filed informed consent for data use upon treatment.

### Participants

Patients with EAC who underwent surgical resection with curative intent between 2001 and 2022 at Heidelberg University Hospital, Germany, CLARUNIS University Center of Gastrointestinal and Liver Diseases, St. Clara and University Hospital of Basel, Switzerland were included. Patients with distant metastases or clinically staged UICC-stage I disease, were excluded^7^. Therefore, only patients with a potential benefit from neoadjuvant therapy according to the ESMO and NCCN-Guidelines were included^5,21^.

### Neoadjuvant treatment

All treatment decisions including indication for neoadjuvant therapy or upfront surgery were made in an interdisciplinary tumor board based on current imaging studies, pathology, and patient status. Board members consisted of experienced upper GI-surgeons, medical oncologists, radiation oncologists, radiologists, and pathologists in all centers. The decision on the treatment plans was made independent of this study. Neoadjuvant (radio)chemotherapy was administered with either four cycles of FLOT (5-fluorouracil, leucovorin, oxaliplatin, docetaxel), platinum-based radio-chemotherapy, or other regimens including EOX and ECF (Supplementary Table 1, Supplemental Digital Content 1, http://links.lww.com/JS9/A974).

### Surgical methods

Surgery was performed by abdominothoracic esophagectomy with Ivor–Lewis reconstruction (ILE) for cancers proximal to the gastroesophageal junction (GEJ) or transhiatal esophagectomy with total gastrectomy and Roux-Y reconstruction (THG) in GEJ Siewert type 3-cancers based on upper endoscopy results^22^. Decision for ILE or THG in GEJ 2-cancers were based on randomization under study conditions or by the surgeon’s preference^23^. Signet ring cell positive cancers with known extend to the stomach were preferably treated with THG. A gastric conduit was routinely used for reconstruction in ILE with a colon conduit as an alternative when reconstruction with the former method was not possible.

### Follow-up

Patients were followed up with regular clinical visits, serologic, radiographic, and endoscopic diagnostics until 60 months postoperatively. Follow-up data were obtained through electrical records of clinical visits, telephone interviews with patients or their primary care provider, and death certificates.

### Outcomes

The AJCC/UICC 8th edition was used for TNM-staging^7^. Treatment response was assessed according to the Becker or Mandard classification^10,24^. Becker 1a and Mandard 1 (complete regression) as well as Becker 1b (<10% vital tumor cells) and Mandard 2 (rare residual cancer cells scattered through the fibrosis) were defined as major histopathologic treatment response. Relative dose intensity (RDI) was calculated by the administered dose divided by the planned dose. OS was defined as the time from diagnosis to death. When the time of diagnosis was not available, the starting date of the first treatment was used. Disease-free survival (DFS) was defined as the time from surgery to cancer recurrence.

### Statistical analysis

The age cut-off for both age groups was set at the upper (56.6 years) and lower quartile (71.3 years) for the old and young subgroup, respectively. Differences in the distribution of categorical data were compared using the *χ* ^2^ test, and continuous data were compared using the Student’s *t*-test. The Kruskal–Wallis test was used for comparisons of multiple groups with nonparametric data. Missing data were mentioned in the tables and removed for group comparisons. Univariate and multivariate Cox proportional hazards regression was used to identify the variables associated with survival benefit. Hazard ratios (HR) and 95% CI were calculated for each pretreatment-assessed variable. Survival comparisons were visualized using the Kaplan–Meier curves. Subgroup analyses were performed for different treatment regimens and arbitrary age cut-offs for early-onset (≤50 years) and late-onset (≥75 years) as used in other studies^17,18^.

Statistical analysis was performed using ʻRʼ statistical software (version 4.2.0). Survival and Survminer packages were used for Kaplan–Meier and Cox regression analyses. Ggplot2 and Forester were used for data visualization. A two-sided *P*-value of <0.05 was considered statistically significant.

## Results

### Study population

**Figure 1:**
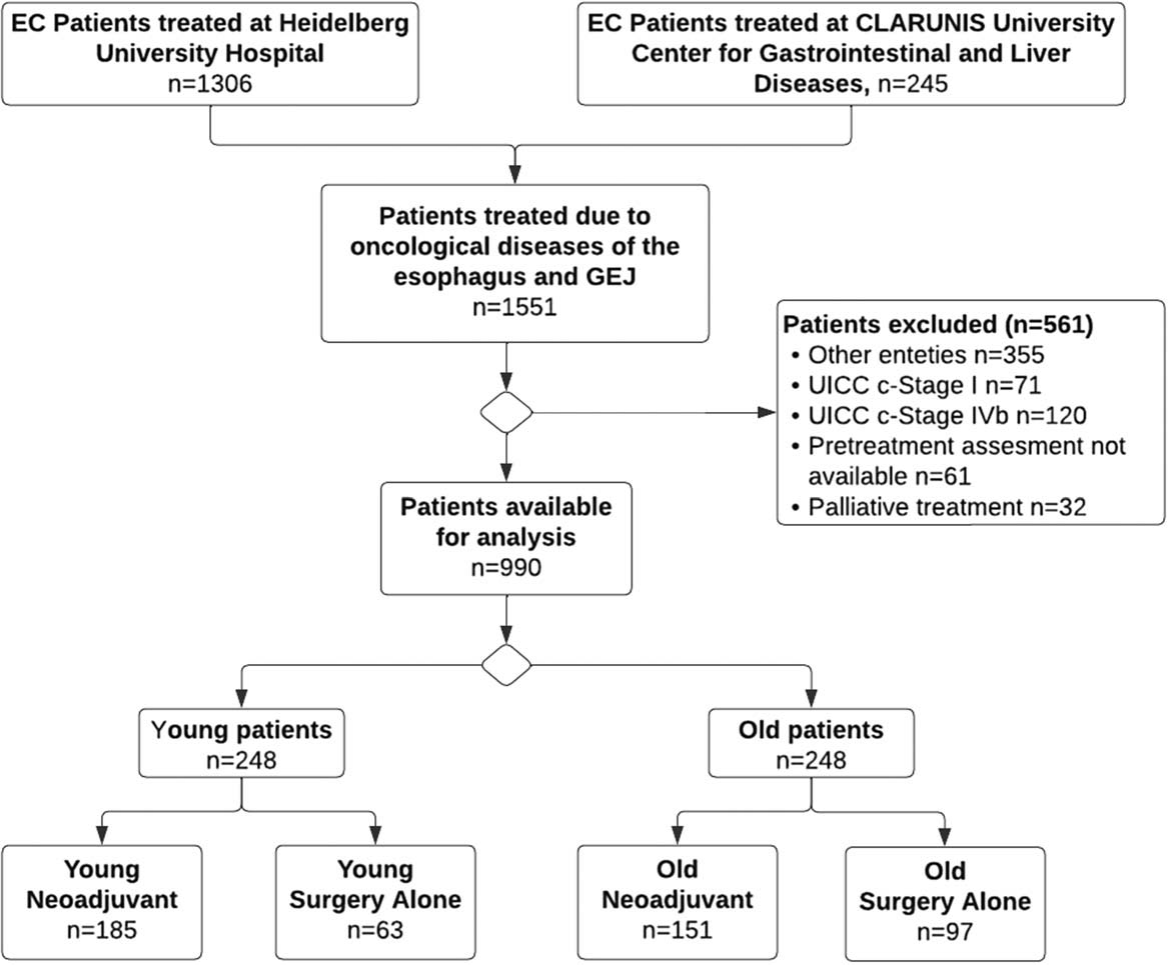
Patient selection. Flowchart of the patient selection. EC, esophageal cancer; GEJ, gastroesophageal junction, exclusion for one or multiple listed reasons, other entities including squamous cell carcinoma, endocrine carcinoma, GIST, operations for recurrent cancer.

A total of 1551 patients were screened of which 990 patients met the inclusion criteria (Fig. 1). The median age was 64.4 years with an interquartile range of 56.6–71.3 years, which also marked the age cut-offs for young and old cohorts. Median follow-up for surviving patients was 39.3 months and 69.8 months for young and 31.1 and 53.8 months for elderly patients with and without neoadjuvant treatment, respectively.

### Baseline characteristics

All baseline characteristics are presented in Table 1. There were more locally advanced tumors and nodal-positive disease within the neoadjuvant treated groups compared to surgery alone. AJCC/UICC 8th Edition cTNM-Stages showed more advanced stages within the neoadjuvant treated groups (III and IVa young: 98.3 vs. 73.3%, *P*<0.001 and old: 98.0 vs. 65.6%, *P*<0.001, Supplementary Table 1, Supplemental Digital Content 1, http://links.lww.com/JS9/A974). Furthermore, according to the ASA-classification, both neoadjuvant treatment groups had higher operative risk profiles and were more often treated with ILE than THG.

**Table 1:** Pretreatment assessment.

	Young			Old			
	Neoadjuvant treatment	Surgery alone	*P*	Neoadjuvant treatment	Surgery alone	*P*	P Young vs. old
Total *N*	185	63		151	97		0.001
Age (years)			0.313			<0.001	<0.001
median (SD)	52.4 (6.27)	49.94 (5.78)		74.64 (3.04)	76.78 (4.52)		
ASA			0.039			0.017	<0.001
1/2	97 (53.3%)	43 (68.3%)		50 (33.3%)	47 (48.5%)		
3/4	85 (46.7%)	20 (31.7%)		100 (66.7%)	50 (51.5%)		
missing	3	0		1	0		
Sex			0.300			0.343	0.159
Female	33 (17.9%)	15 (23.8%)		34 (22.5%)	27 (27.8%)		
Male	152 (82.2%)	48 (76.2%)		117 (77.5%)	70 (72.2%)		
Tumor-Location			0.449			0.002	0.312
GEJ 1	87 (47.0%)	24 (38.1%)		73 (48.3%)	42 (43.3%)		
GEJ 2	81 (43.8%)	33 (55.4%)		51 (33.8%)	50 (51.5%)		
GEJ 3	17 (9.2)	6 (9.5%)		27 (17.9%)	5 (5.2%)		
cT-Stage			<0.001			<0.001	0.049
cT1	3 (1.7%)	0 (0%)		0 (0%)	1 (1.1%)		
cT2	11 (6.1%)	19 (31.1%)		10 (6.8%)	39 (41.1%)		
cT3	146 (80.7%)	39 (63.9%)		123 (83.7%)	54 (56.8%)		
cT4	21 (11.6%)	3 (4.9%)		14 (9.5%)	1 (1.1%)		
cTx	4	2		4	2		
cN-Stage			<0.001			<0.001	0.524
cN-	22 (11.9%)	41 (66.1%)		17 (11.3%)	52 (54.7%)		
cN+	163 (88.1%)	21 (33.9%)		134 (88.7%)	43 (45.3%)		
cNx	0	1		0	2		
Tumor Grade							
G1	7 (4.3%)	3 (4.8%)	0.612	2 (1.5%)	4 (4.2%)	0.126	0.584
G2	68 (42.0%)	21 (33.3%)		59 (45.0%)	32 (33.3%)		
G3 + 4	87 (53.7%)	39 (61.9%)		70 (53.4%)	60 (62.5%)		
missing	22	0		20	1		
Type of neoadjuvant therapy							0.121
FLOT	105 (56.8%)		79 (52.3%)				
CT other	51 (27.6%)			35 (23.2%)			
RCT	29 (15.7%)			37 (24.5%)			
Type of Surgery			0.006			0.006	0.031
ILE	137 (74.1%)	35 (55.6%)		101 (66.9%)	48 (49.5%)		
THG	48 (25.9%)	28 (44.4%)		50 (33.1%)	49 (50.5%)		

Data are *N* (%) or median (SD).

ASA, American Society of Anesthesiologists Classification System; CT, chemotherapy; FLOT, 5-fluorouracil, leucovorin, oxaliplatin, docetaxel; GEJ, gastroesophageal junction (Siewert-classification); GEJ 1, gastroesophageal junction siewert Type 1 and above; ILE, abdominothoracic esophagectomy with Ivor–Lewis reconstruction; RCT, radio-chemotherapy; THG, transhiatal extended gastrectomy.

There were no significant differences within the group comparisons of both surgery alone groups except for age. Also, except for age, ASA status, and tumor location, there were no significant differences when comparing both neoadjuvant treated groups (Supplementary Table 2, Supplemental Digital Content 1, http://links.lww.com/JS9/A974).

### Neoadjuvant treatment and further cytotoxic treatments

Neoadjuvant treatment was administered to 185 out of 248 (74.2%) young patients and 151 out of 248 (60.9%) elderly patients (*P*=0.001). The FLOT-regimen was the most used neoadjuvant treatment in both groups (young: 56.8 vs. old: 52.3%, *P*=0.121). Other chemotherapy regimens (Supplementary Table 1, Supplemental Digital Content 1, http://links.lww.com/JS9/A974) were used in 51 (27.6%) young and 35 (23.2%) elderly patients. Radio-chemotherapy was administered in 29 (15.7%) young and 37 (24.5%) elderly patients. Neoadjuvant treatment was discontinued in 11 (5.9%) young versus 21 (13.9%) elderly patients (*P*=0.013). In young patients, the main reason for discontinuation was nonresponse (63.6%), whereas toxicity with severe side effects was the main reason within the elderly cohort (76.2%). In total, 23 (12.4%) of young patients and 61 (40.4%) old patients did not receive the full dose (*P*<0.001). Detailed results are presented in Table 2.

**Table 2:** Neoadjuvant treatment received.

	Young *N*=185	Old *N*=151	*P*
Discontinuation of neoadjuvant treatment	11 (5.9%)	21 (13.9%)	0.013
Reason for discontinuation			0.021
Noncompliance	0 (0%)	1 (4.8%)	
Nonresponse	7 (63.6%)	4 (19.0%)	
Toxicity	4 (36.4%)	16 (76.2%)	
Dose reduction	23 (12.4%)	61 (40.4%)	<0.001
Reason for dose reduction			0.013
Reduced tolerability	2 (16.7%)	23 (57.5%)	
Severe side effects	10 (83.3%)	17 (42.5%)	

Data are *n* (%), Pearson’s *χ* ^2^ test; Fisher’s exact test, Reason for dose reduction excludes patients with discontinuation of treatment.

Adjuvant treatment was administered in 67.7% of the neoadjuvant young group and 45.0% in the surgery alone group (*P*=0.002). The same was observed with old patients being treated more often with adjuvant chemotherapy when neoadjuvantly treated (38.9 vs. 15.0%, *P*<0.001). Treatment of recurrent cancer was performed for 90.1 vs. 81.3% young patients (*P*=0.209) and 89.7 vs. 45.5% elderly patients (*P*<0.001, Table 3) with diagnosed recurrence.

**Table 3:** Postoperative assessment.

	Young			Old			
	Neoadjuvant treatment	Surgery alone	*P*	Neoadjuvant treatment	Surgery alone	*P*	Young vs. old
Total *N*	185	63		151	97		
pT-Stage			0.007			<0.001	0.497
0	20 (10.8%)	0 (0%)		18 (11.9%)	0 (0%)		
1	22 (11.9%)	7 (11.1%)		21 (13.9%)	19 (19.6%)		
2	33 (17.8%)	10 (15.9%)		15 (9.9%)	20 (20.6%)		
3	99 (53.5%)	35 (55.6%)		91 (60.3%)	47 (48.5%)		
4	11 (5.9%)	11 (17.5%)		6 (4.0%)	11 (11.3%)		
pN-Stage			0.007			0.041	0.012
0	78 (42.2%)	18 (28.6%)		71 (47.0%)	34 (35.1%)		
1	40 (21.6%)	9 (14.3%)		38 (25.2%)	20 (20.6%)		
2	32 (17.3%)	11 (17.5%)		17 (11.3%)	22 (22.7%)		
3	35 (18.9%)	25 (39.7%)		25 (16.6%)	21 (21.6%)		
Resection-Margins			0.106			0.072	0.311
R0	169 (91.4%)	53 (84.1%)		141 (93.4%)	84 (86.6%)		
R1	16 (8.6%)	10 (15.7%)		10 (6.6%)	13 (13.4%)		
Tumor regression grade							0.286
Major	55 (30.7%)			52 (36.4%)			
Minor	124 (69.3%)			91 (63.6%)			
Missing	3			7			
Resected lymph nodes			0.114			0.096	0.052
Median (SD)	26 (11.00)	23 (11.82)		24 (10.02)	22 (8.21)		
Complication			0.384			0.352	0.246
No	134 (72.4%)	42 (66.7%)		94 (62.3%)	66 (68.0%)		
Yes	51 (27.6%)	21 (33.3%)		57 (37.7%)	31 (32.0%)		
30-day mortality			0.425			0.294	0.011
Yes	1 (0.5%)	1 (1.6%)		5 (3.4%)	6 (6.2%)		
90-day mortality			0.449			0.92	0.001
Yes	3 (1.6%)	2 (3.2%)		13 (8.6%)	8 (8.2%)		
Adjuvant treatment			0.002			<0.001	<0.001
No	54 (32.3%)	33 (55.0%)		80 (61.1%)	68 (85.0%)		
Yes	113 (67.7%)	27 (45.0%)		51 (38.9%)	12 (15.0%)		
Missing	18	3		20	17		
Treatment of recurrence			0.209			<0.001	0.027
No	7 (9.9%)	6 (18.7%)		4 (10.3%)	12 (54.5%)		
Yes	64 (90.1%)	26 (81.3%)		35 (89.7%)	10 (45.5%)		
Missing	17	5		13	16		

Data are *N* (%) or median (SD), Major pathohistological response=Becker classification, 1A, 1B and Mandard-TRG-1 and 2; Complication=Clavien–Dindo 3A or higher.

### Postoperative outcomes

Postoperative outcomes are presented in Table 3. Notably, there was no significant difference in major histopathologic treatment response within the neoadjuvant treatment groups (young 30.7 vs. old 36.4%, *P*=0.286). Histopathologic workup showed more nodal-negative disease and lower pT-Stages in both neoadjuvant treatment groups versus surgery alone. Histopathologic surrogate markers, such as tumor free resection-margins and suffering major postoperative complications were equally distributed in all group comparisons. Analysis of all patients showed neoadjuvant treated patients had higher rates of R0-resections (90.4 vs. 85.1%, *P*=0.015).

Younger patients had lower 30-day and 90-day mortality but did not differ within both young versus old group comparisons. Sixty-seven (89.3%) of the reported deaths in young patients but only 60 (63.2%) of deaths in elderly patients were cancer related (*P*<0.001, Supplementary Table 1, Supplemental Digital Content 1, http://links.lww.com/JS9/A974).

### Survival analysis

**Figure 2:**
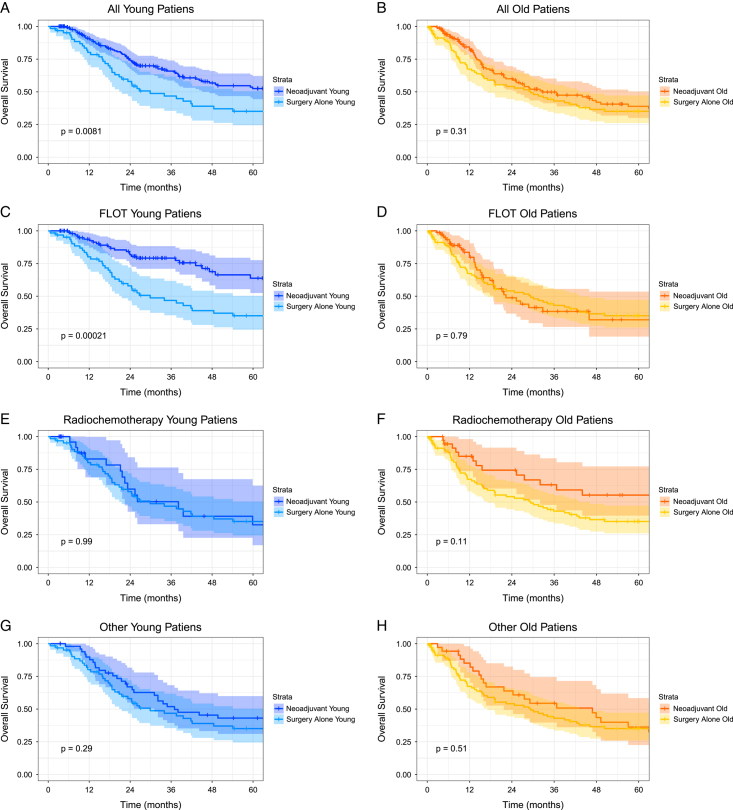
Kaplan–Meier Survival Curves. Kaplan–Meier estimates of overall survival according to age groups for all patients (A and B), patients treated with FLOT (C and D), patients treated with radio-chemotherapy (E and F), or patients treated with other than the aforementioned treatment regimens (G and H) compared to surgery alone. Only for young patients and the subgroup of young patients receiving FLOT there is a demonstrated survival benefit compared to upfront resection. *P*-value calculated by log-rank test, *Y*-Axis=Survival Probability, + represents censored patients at last follow-up.

Young patients showed a significant OS benefit when treated with neoadjuvant therapy compared to surgery alone (median OS: 85.6 vs. 29.9 months, HR 0.62, 95% CI: 0.42–0.92, Fig. 2A). In contrast, elderly patients did not significantly benefit from neoadjuvant treatment (median OS: neoadjuvant 32.8 vs. surgery alone 29.3 months, HR 0.89, 95% CI: 0.63–1.27, Fig. 2B). Three-year survival was 65.4 vs. 48.5% in the young (*P*=0.015) and 48.8 vs. 43.2% in the old cohort (*P*=0.798). Neoadjuvant treatment was not significantly associated with improved DFS in young (median DFS: neoadjuvant 33.4 months vs. surgery alone 22.7 months, HR 0.86, 95% CI: 0.59–1.26, *P*=0.49) or elderly patients (median DFS: neoadjuvant 34.0 months vs. surgery alone 57.4 months, HR 1.04 95% CI: 0.69–1.59, *P*=0.79, Supplementary Figure 1a–b, Supplemental Digital Content 1, http://links.lww.com/JS9/A974). Overall, young patients had better OS (49.7 vs. 32.0 months, *P*<0.001) but similar DFS (30.2 vs. 43.4 months, *P*=0.24) compared to elderly patients.

**Figure 3:**
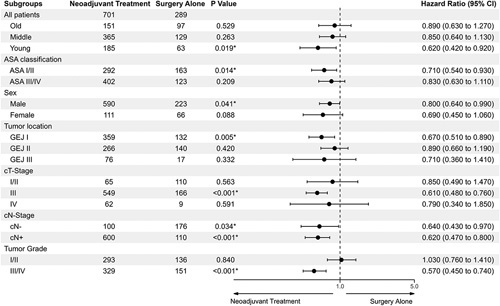
Forrest Plot of the Effect of Neoadjuvant Treatment on the Hazard Ratio for Death. Effect estimates are calculated with univariate Cox Regression analysis for overall survival, the hazard ratio and significant *P*-values (<0.05) for this analysis suggest a survival benefit for young patients (age <56.6 years), American Society of Anaesthesiologists physical status I and II (ASA), male sex, Gastroesophageal Junction (GEJ) Siewert-classification I and above, AJCC-cTNM Stage III, both clinically nodal-positive (cN+) and negative (cN-) disease, and poor tumor differentiation (tumor grade III/IV). Confidence intervals are indicated by horizontal lines. The position of each dot indicates the estimate of benefit form neoadjuvant treatment or surgery alone. Age middle = between 1st and 3rd quartile (age 56.6–71.3 years); Old = older than 71.3 years.

Additionally to the young age, the univariate Cox regression analysis of the subgroups based on pretreatment-assessed values did show a significant OS benefit from neoadjuvant treatment compared to surgery alone in the following subgroups: ASA1/2 (HR 0.71, 95% CI: 0.54–0.93, *P*=0.014), more proximal cancer (EAC and GEJ I: HR 0.67, 95% CI: 0.51–0.89, *P*=0.005), clinical T-stage 3 (HR 0.61, 95% CI: 0.48–0.76, *P*<0.001), clinically nodal-negative disease (cN-: HR 0.64, 95% CI: 0.43–0.97, *P*=0.034), nodal-positive disease (cN+: HR 0.62, 95% CI: 0.47–0.80, *P*<0.001), and poorly differentiated tumors (G3/4: HR 0.57, 95% CI: 0.45–0.74, *P*<0.001, Fig. 3). A multivariate analysis of the aforementioned valuables yielded significant OS benefits for all subgroups except for well-differentiated neoplasms (HR 0.77, 95% CI: 0.55–1.08, *P*=0.130), cT1 or 2 (HR 0.68, 95% CI: 0.36–128, *P*=0.233) and cT4-Stages (HR 0.58, 95% CI: 0.24–1.38, *P*=0.217, Supplementary Figure 2, Supplemental Digital Content 1, http://links.lww.com/JS9/A974). Mentionable, elderly patients did benefit from neoadjuvant treatment in the multivariate analysis as well (HR 0.55, 95% CI: 0.37–0.81, *P*=0.003).

Adjuvant treatment was associated with a significant OS benefit in elderly patients (no adjuvant: 28.9 months vs. adjuvant: 46.9 months, HR 0.63, 95% CI: 0.41–0.97, *P*=0.035, Supplementary Figure 3, Supplemental Digital Content 1, http://links.lww.com/JS9/A974) but not in young patients (49.0 months vs. 59.2 months, HR 0.98, 95% CI: 0.65–1.5, *P*=0.917). There was no significant effect of adjuvant treatment on DFS (young: HR 0.97, 95% CI: 0.67–1.40, *P*=0.88, old: HR 0.98, 95% CI: 0.82–1.2, *P*=0.43).

Except for cT3-Tumors (HR 0.73, 95% CI: 0.57–0.93, *P*=0.01), no subgroup was associated with a DFS benefit in the univariate analysis (Supplementary Figure 4, Supplemental Digital Content 1, http://links.lww.com/JS9/A974). Multivariate analysis of DFS showed significant values for the benefit of neoadjuvant treatment in ASA III/IV, male patients, GEJ 1, cT-Stage 3, both cN- and cN+, and poorly differentiated tumors (Supplementary Figure 5, Supplemental Digital Content 1, http://links.lww.com/JS9/A974).

### Subgroup analyses

#### Type of treatment

Dose reduction for elderly patients was seen more often in FLOT treatment compared to radio-chemotherapy (57 vs. 5.4%, *P*<0.001, Supplementary Table 4, Supplemental Digital Content 1, http://links.lww.com/JS9/A974). For FLOT treated patients, young patients received a significantly higher mean relative dose intensity (RDI: 0.98 (SD 0.07) vs. 0.84 (SD 0.16,) *P*<0.001) and lower rates of RDI less than or equal to 0.80 (5.7 vs. 37.9%, *P*<0.001, Supplementary Table 5, Supplemental Digital Content 1, http://links.lww.com/JS9/A974). The effect of OS benefit was even more pronounced in the young subgroup receiving FLOT (multivariate: HR 0.35, 95% CI: 0.20–0.62, *P*<0.001, Supplementary Figures 6, Supplemental Digital Content 1, http://links.lww.com/JS9/A974 and 7, Supplemental Digital Content 1, http://links.lww.com/JS9/A974), whereas the OS benefit within elderly was limited also in the multivariate analysis (HR 0.62, 95% CI: 0.38–1.02, *P*=0.059). Similar effects were present for DFS (multivariate young: HR 0.62, 95% CI: 0.38–1.03, *P*=0.064 and old: HR 0.82, 95% CI: 0.45–1.50, *P*=0.516, Supplementary Figures 8, Supplemental Digital Content 1, http://links.lww.com/JS9/A974 and 9, Supplemental Digital Content 1, http://links.lww.com/JS9/A974). A significant benefit for DFS was only seen in patients with poorly differentiated tumors (HR 0.68, 95% CI: 0.46–0.99, *P*=0.044). For other treatment regimens, no multivariate analyses can be performed due to limited numbers. Treatment effects on OS are shown in Figure 2 and DFS in Supplementary Figure 1 (Supplemental Digital Content 1, http://links.lww.com/JS9/A974). Although insignificant, elderly patients benefitted more from radio-chemotherapy compared to FLOT (HR 0.93, 95% CI: 0.85–1.02, *P*=0.108). For young patients, the opposite effect was shown (HR 1.14, 95% CI: 1.04–1.25, *P*=0.005).

### Traditional age cut-offs for early versus late-onset EAC

Using traditional cut-offs for early-onset (≤50 years) and late-onset (≥75 years) showed similar effects on overall and DFS. A significant benefit was only seen for OS in patients aged 50 or below (not reached versus 34.0 months, *P*=0.031). Detailed results are shown in Supplementary Table 6 (Supplemental Digital Content 1, http://links.lww.com/JS9/A974).

## Discussion

In contrast to other gastrointestinal malignancies, young age was associated with improved overall and similar DFS in this study. Neoadjuvant treatment was associated with a highly relevant median survival benefit in the young cohort, whereas the median gain in OS in the old cohort was a mere three months. Old patients did not experience an OS benefit or DFS in the univariate analysis. Next to young age, an OS benefit was only observed in patients with lower ASA scores and poorly differentiated tumors. When corrected for pretreatment clinically assessed tumor characteristics and baseline characteristics in the multivariate analysis, both younger and older patients show a benefit from neoadjuvant treatment. However, the difference in median OS clearly indicates a stronger benefit of neoadjuvant therapy in young compared to elderly patients. This is highlighted by a 17% 3-year survival benefit after neoadjuvant treatment in young patients but only by a 5% 3-year survival difference in elderly patients. These treatment effects are pronounced in the subgroup analysis of FLOT treatment. When the subgroup of patients with neoadjuvant radio-chemotherapy was analyzed, elderly patients had better tolerance to treatment without impairment of survival outcomes. These results suggest that radio-chemotherapy may be the better treatment option for elderly patients. Despite these highly clinically relevant survival differences, there were no differences in major histopathologic response to neoadjuvant treatment between the young and old age groups. Further highlighting the issues of potential toxicity of neoadjuvant treatment in the elderly patients is the significantly higher rate of dose reduction or early termination of neoadjuvant therapy caused by severe side effects.

As it can be expected in a retrospective study, patients treated with upfront surgery had significantly lower cT-stages and less clinically node-positive disease compared to both neoadjuvant treated age subgroups since the current guidelines indicate that patients with nodal-positive disease benefit most from neoadjuvant treatment^5,21^. Existing guidelines do not consider variables such as patient or tumor characteristics like fitness level and tumor grade, which are important factors in treatment decision-making and may be associated with age^5,21^. However, high-quality evidence for neoadjuvant treatment within the elderly is lacking as the analysis of the treatment effect on long-term outcomes of the CROSS trial did not include patients older than 75 years. Its survival analyses only showed a significant treatment benefit in patients with a WHO performance score of 0, male sex, and node-negative disease^25^. Furthermore, the benefit was pronounced to squamous cell carcinoma rather than adenocarcinoma, which is the most treated esophageal malignancy within western countries. Similarly, the MAGIC trial only showed limited evidence in its subgroup analysis and elderly patients were also underrepresented^14^. Therefore, it is unclear which subgroups, especially within elderly patients really benefit from neoadjuvant therapy and which may benefit more from upfront surgery. Thus, patient selection for individualized treatment remains challenging. This analysis shows a similar benefit for nodal-negative and nodal-positive disease in the univariate and multivariate analysis. This finding can be explained by imprecise clinical staging. Sixty percent of the cT2 and clinically staged nodal-negative disease show nodal-positive disease in the histopathologic workup despite neoadjuvant treatment^26,27^. Furthermore, there is a difference of about 30% in cN-negative to pN-negative in the surgery alone groups. Pathologic N-negativity was even more present in both neoadjuvant treated groups despite limited histopathological response. This finding is in line with other studies and indicates that cN-stage staging cannot be relied upon for treatment decisions^26,27^.

This is the first clinical analysis showing the effects of neoadjuvant therapy in elderly patients. In this study, we found a clinically significant OS benefit for neoadjuvant therapy only in younger patients whereas elderly patients only insignificantly gained a median OS of 3.5 months and 5% OS after 3 years. The price for this meager benefit was a dose reduction or abortion of neoadjuvant therapy in two out of five of the elderly patients. Especially for FLOT treatment, tolerability in elderly patients is questionable. Also, the analysis of RDI reveals that due to these severe side effects, a substantial number of elderly patients treated with FLOT did not receive sufficient doses (RDI ≤0.8)^28^. Radio-chemotherapy seems to be tolerated better by elderly patients. Since only resected patients were analyzed, it remains unclear how many patients were excluded from surgery due to the severe side effects of neoadjuvant therapy. Evidence shows that in real world and without a strict preselection of patients in RCTs approximately one out of six patients who are started on neoadjuvant therapy will not make it to surgery due to disease progression or poor general condition^29^. Inclusion of those patients would have worsened OS in neoadjuvant treated patients, especially within the older cohort. Although the multivariate analysis showed that elderly patients benefit from neoadjuvant treatment statistically, the clinical benefit is strongly limited with a gain in OS that equals approximately the length of neoadjuvant treatment itself. Given the difference in the univariate and multivariate analysis, appropriate patient selection for neoadjuvant treatment is crucial. These results suggest that some elderly patients may benefit from upfront surgery.

As seen in hallmark studies, an improved rate of R0 resection due to neoadjuvant treatment was seen in the whole population^14,25^. Although not significant, DFS was better within the multivariate analysis for young patients when treated with FLOT. However, there was no observed difference in DFS in elderly patients. Non cancer related deaths may not only decreased power in the DFS-analysis but also contribute to a decreased potential of treatment benefit within the elderly cohort. Due to the higher rate of dose reduction, a lower rate of adjuvant treatment, and less treatment of recurrence within the old group it is possible, that chemotherapy did not live up to its full potential. Since administration of adjuvant and recurrence therapy was lower in the upfront surgery group and in regard of the survival results, different efficacies of chemotherapy due to age is possible. A part of this effect may be explained by chemotherapy-induced accelerated immunosenescence in elderly patients^30^. Translational studies show that CD8^+^ and CD3^+^effector T cell populations decrease during the course of chemotherapy, leading to an accelerated aging and less suppressing TME over time^30–32^. A benefit of OS for elderly patients can be explained with being a surrogate marker for the patient’s condition after surgery^33^. The limited benefit of adjuvant treatment for DFS for all patients and OS in young patients is in line with previous analyses that investigated the role of adjuvant after neoadjuvant treatment and highlights the need for defining the optimal treatment sequence^33–35^.

In line with the current evidence based on the FLOT4 trial and others, the FLOT-regimen resulted in more pronounced benefit of neoadjuvant therapy, especially in the younger patients^3,36^. Given their better tolerability of treatment, future studies on more effective treatment regimens are expected to improve the low major histopathologic response rate especially within young patients. Major histopathologic response to neoadjuvant treatment in the absence of tumor involvement of regional lymph nodes is an important prognostic surrogate marker in EAC^9^. The low response rate of approximately one out of three patients could be due to a lack of molecular insight when treatment decisions are made. Molecular alterations such as SMARCA4 are associated with less benefit to neoadjuvant therapy^37^. More studies are needed to assess the role of age in the approach of molecular guided therapy. Recently, the CheckMate577 study has shown a significant survival benefit from nivolumab treatment (PD1-checkpoint inhibitor) in an adjuvant setting^38^. Younger patients may benefit more from this additional treatment in future as according to a mouse model they have more effects of PD-L1 monoclonal antibodies and have more tolerability to undergo additional aggressive treatments^39,40^. Further studies should investigate whether tumor biology and the properties of the tumor microenvironment are different depending on age.

## Limitations

As this was a retrospective analysis, it is associated with potential bias associated with its study design. Selection bias may be present as neoadjuvant treated patients with progress to unresectable disease where not included, making this study a treatment received analysis. In the highly preselected patient cohorts of the CROSS and FLOT trials, the proportion of progress to unresectable disease was 4–5%, but this drawback was compensated by better resectability compared to the surgery alone group^3,25^. Inclusion of these patients may would have influenced the treatment effects shown. Furthermore, there is a selection bias to more advanced disease in the neoadjuvant treated groups. However, since both the neoadjuvant treated groups and the surgery alone groups were comparable, the observed effects of neoadjuvant therapy remain valid. Subanalyses of traditional arbitrary cut-offs of early (≤50 years) and late-onset (≥75 years) disease did show similar treatment effects. Also, the selection of neoadjuvant therapy was based on institutional preference for FLOT in one center and radio-chemotherapy in the other centers. Due to the limited number of patients treated with radio-chemotherapy or chemotherapeutic treatment regimens other than FLOT, a multivariate subanalysis was only possible for the latter treatment regimen. Due to the same reason, no definitive statement can be made to define optimal treatment regimens stratified to age. Furthermore, the treatment decision of the interdisciplinary tumor board was based on current guidelines. Change of guidelines during the study period may affects results. Before the MAGIC study was published in 2006, patients were treated with surgery alone^14^. This leads to a potential length time bias due to shorter follow-ups in the neoadjuvant groups and a suppression of technical aspects improving the prognosis of esophageal cancer patients. Confounding variables associated with age may have been underestimated. For example, the ASA score has inherent subjectivity in scoring and shows moderate inter-rater reliability^41^. The effects of neoadjuvant treatment may have influenced ASA scoring preoperatively. Therefore, treatment decisions should not be based on age and tumor stage alone but should always be individualized according to available evidence.

## Conclusion

This analysis shows that old patients benefit less from neoadjuvant treatment compared to younger patients in terms of gain of median survival. They also experience more side effects requiring dose reduction. Therefore, it seems likely that a substantial proportion of elderly patients do not benefit from neoadjuvant treatment while experiencing relevant side effects reducing the quality of life. Therefore, upfront surgery should be preferred in elderly patients with low potential benefit of neoadjuvant treatment. If a neoadjuvant therapy is chosen in elderly patients, radio-chemotherapy seems to be better tolerated in the elderly patients with similar efficacy. However, current clinically assessed oncologic surrogate markers especially cN-Stage do not reliably predict the long-term benefits of neoadjuvant therapy. Further studies are needed to assess the role of age and tumor biology to improve patient selection to neoadjuvant treatment, especially within elderly patients.

## Ethical approval

Ethical approval for this study (Ethical Committee N° S-2-649-2012) was provided by the Ethical Committee of Heidelberg University, Heidelberg, Germany on 01 February 2012.

## Consent

Written informed consent was obtained from the patient for publication and any accompanying images. A copy of the written consent is available for review by the Editor-in-Chief of this journal on request.

## Sources of funding

No funding was received for this project.

## Author contribution

I.F.R., A.T.B., H.N., and B.P.M.: conceptualization; N.C., K.N., L.F., and L.S.: data curation; B.J.M.vdW. and I.F.R.: formal analysis; funding acquisition: N/A; I.F.R., H.N., and A.T.B.: investigation; B.J.M.vdW., I.F.R., and A.T.B.: methodology; B.P.M.: project administration and resources; N.C. and I.F.R.: software; A.T.B. and H.N.: supervision; H.N. and N.C.: validation; I.F.R. and N.C.: visualization; I.F.R.: writing original draft. All authors contributed in writing – review and editing.

## Conflicts of interest disclosure

There are no conflicts of interest for any authors.

## Research registration unique identifying number (UIN)

The study protocol was published on the research registry (researchregistry9273).

## Guarantor

Beat P. Müller-Stich.

## Data availability statement

The data provided here is accurate to the best of our knowledge.

## Provenance and peer review

No invite.

## Presentations

This study was presented at the 29th Annual Meeting of the European Surgical Association, 12–13 May 2023, Bordeaux, France.

## Supplementary Material

SUPPLEMENTARY MATERIAL
