# Supplementary material for: Age-dependent benefit of neoadjuvant treatment in adenocarcinoma of the esophagus and gastroesophageal junction: a multicenter retrospective observational study of young versus old patients
Source: Int J Surg. 2023 Sep 14;109(12):3804–14. doi: 10.1097/JS9.0000000000000713 (PMC10720874; doi:10.1097/JS9.0000000000000713)

SUPPLEMENTARY MATERIAL

AGE DEPENDENT BENEFIT OF NEOADJUVANT TREATMENT IN ADENOCARCINOMA OF THE ESOPHAGUS AND GASTROESOPHAGEAL JUNCTION – A MULTICENTER RETROSPECTIVE OBSERVATIONAL STUDY OF YOUNG VERSUS OLD PATIENTS

Table of Contents

Figure 1: Kaplan Meier Disease-Free Survival 2

Figure 2: Multivariate Cox-Regression Analysis Overall Survival 3

Figure 3: Kaplan Meier Survival Curve for Elderly Patients Adjuvant Treatment 4

Figure 4: Univariate Disease-Free Survival Forest Plot 5

Figure 5: Multivariate Disease-Free Survival Forest Plot 5

Figure 6: FLOT-Subanalysis OS-univariate 6

Figure 7: FLOT-Subanalysis OS-multivariate 6

Figure 8: FLOT-Subanalysis DFS-univariate 7

Figure 9: FLOT-Subanalysis DFS-multivariate 7

Table 1: Other Values Age comparisons 8

Table 2: Neoadjuvant Young vs. Old and Upfront Surgery Young vs. Old 9

Table 3: Neoadjuvant Treatment Received subanalyses 10

Table 5: Relative Dose intensity 10

Table 6: Traditional Age cut-offs Early-onset (<50) and Late-onset (>75) 11

Figure 1: Kaplan Meier Disease-Free Survival

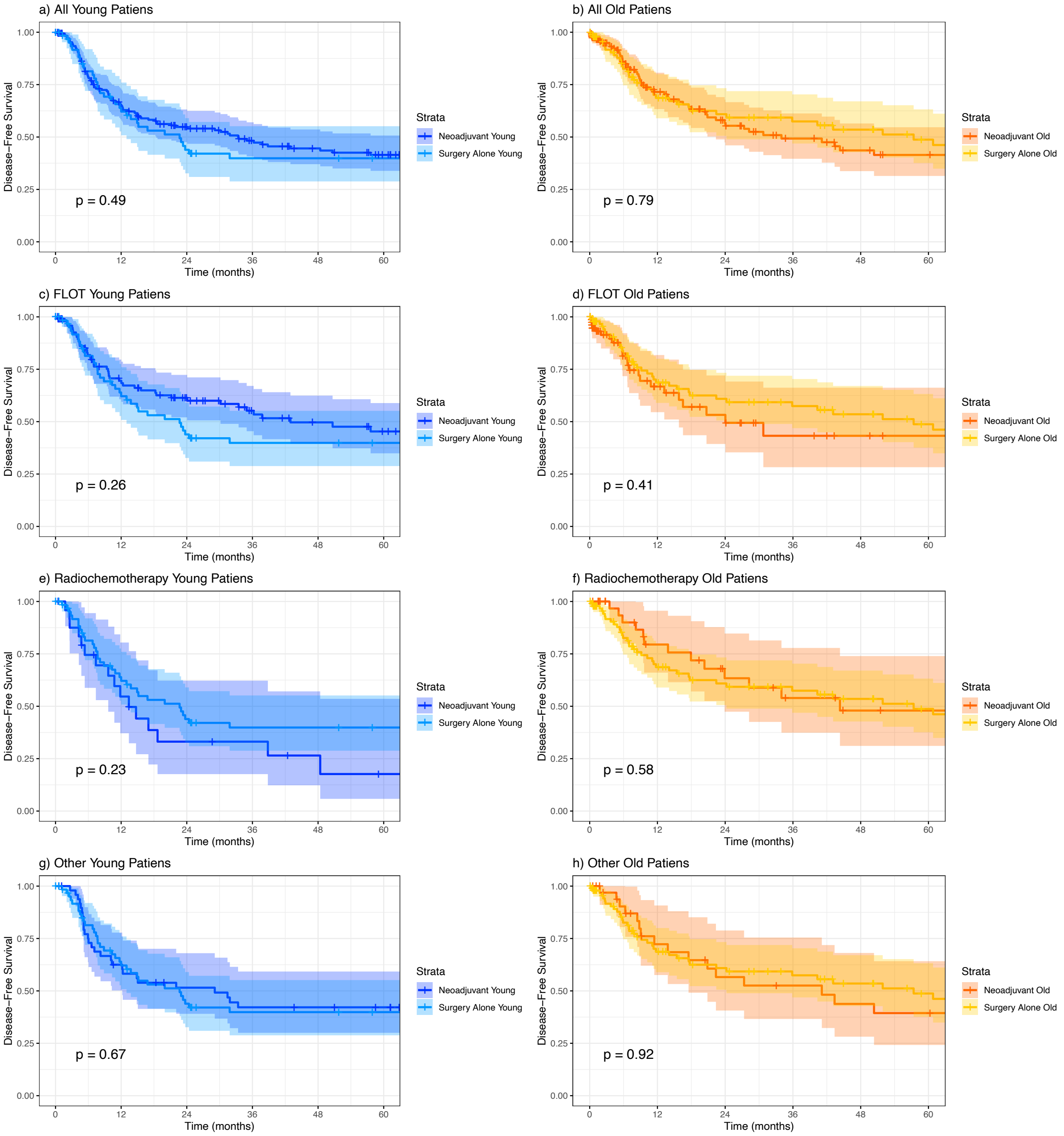

Legend: P-value calculated by log-rank test, Y-Axis=Disease-Free Survival Probability

Figure 2: Multivariate Cox-Regression Analysis Overall Survival

Subgroups	Neoadjuvant Treatment	Surgery Alone	P Value	Hazard Ratio (95% CI)
All patients	701	289		
Old	151	97	0.003*	0.550 (0.370 to 0.810)
Middle	365	129	0.002*	0.600 (0.440 to 0.820)
Young	185	63	0.001*	0.490 (0.310 to 0.760)
ASA classification				
ASA I/II	292	163	<0.001*	0.520 (0.390 to 0.710)
ASA III/IV	402	123	<0.001*	0.590 (0.430 to 0.820)
Sex				
Male	590	223	<0.001*	0.570 (0.450 to 0.740)
Female	111	66	0.003*	0.490 (0.310 to 0.780)
Tumor location				
GEJ I	359	132	<0.001*	0.510 (0.370 to 0.700)
GEJ II	266	140	0.014*	0.660 (0.470 to 0.920)
GEJ III	76	17	0.009*	0.390 (0.190 to 0.790)
cT-Stage				
I/II	65	110	0.233	0.680 (0.360 to 1.280)
III	549	166	<0.001	0.540 (0.420 to 0.700)
IV	62	9	0.217	0.580 (0.240 to 1.380)
cN-Stage				
cN-	100	176	0.001*	0.500 (0.320 to 0.770)
cN+	600	110	<0.001*	0.580 (0.440 to 0.770)
Tumor Grade				
I/II	293	136	0.130	0.770 (0.550 to 1.080)
III/IV	329	151	<0.001*	0.450 (0.340 to 0.590)

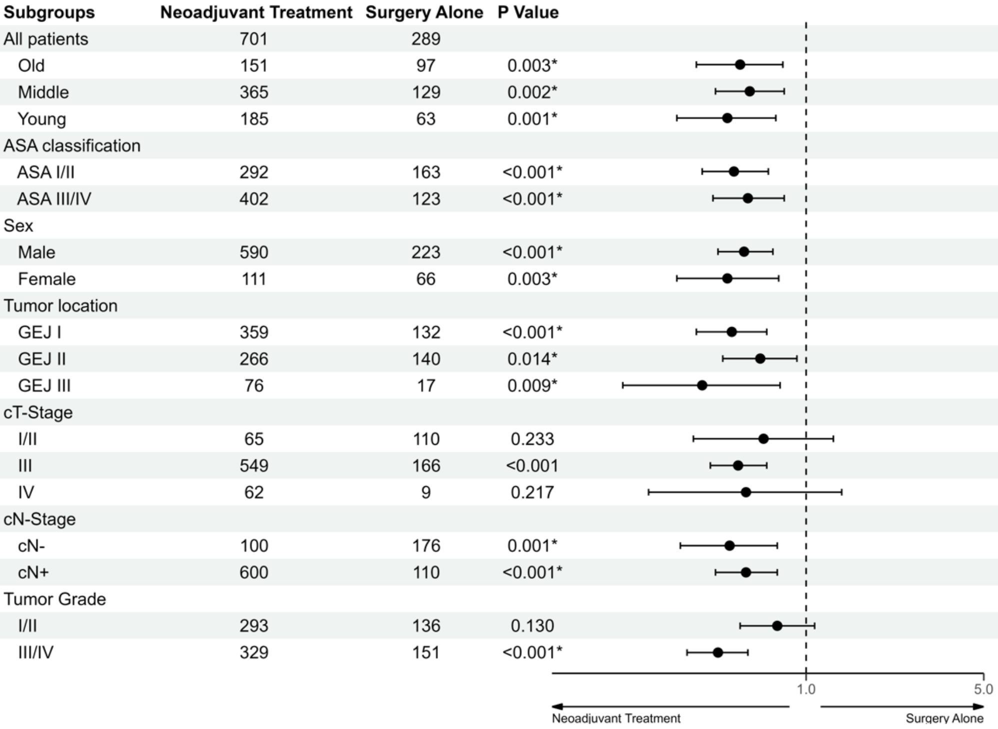

Legend Supplementary Material Tables 2 and 4-9: Middle = between 1st and 3rd quartile (age 56.60-71.29 years); ASA= American Society of Anaesthesiologists physical status; GEJ=Gastroesophageal Junction (Siewert classification), GEJ1= Siewert I and above

Figure 3: Kaplan Meier Survival Curve for Elderly Patients Adjuvant Treatment

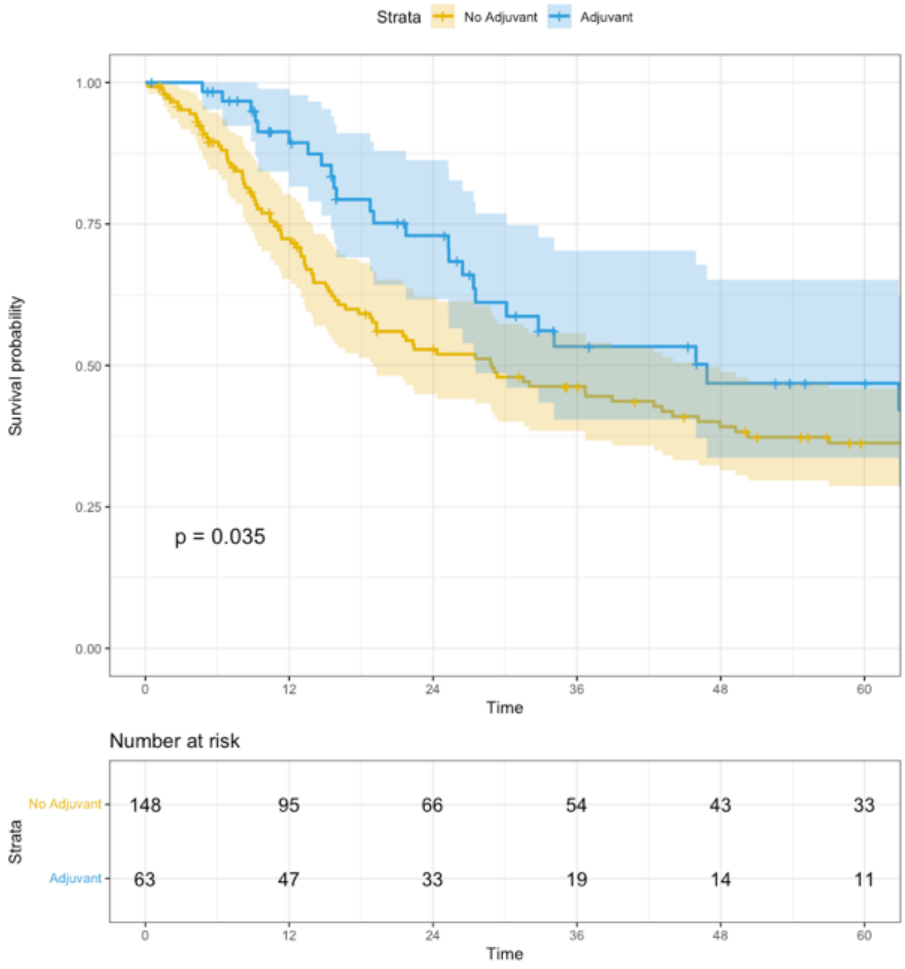

Legend: P-value calculated by log-rank test, Y-Axis=Survival Probability

Figure 4: Univariate Disease-Free Survival Forest Plot

Subgroups	Neoadjuvant Treatment	Surgery Alone	P Value	Hazard Ratio (95% CI)
All patients	701	289		
Old	151	97	0.837	1.040 (0.690 to 1.590)
Middle	365	129	0.519	1.110 (0.810 to 1.530)
Young	185	63	0.444	0.860 (0.590 to 1.260)
ASA classification				
ASA I/II	292	163	0.917	1.020 (0.760 to 1.360)
ASA III/IV	402	123	0.874	1.030 (0.750 to 1.400)
Sex				
Male	590	223	0.761	1.040 (0.820 to 1.310)
Female	111	66	0.904	0.970 (0.600 to 1.570)
Tumor location				
GEJ I	359	132	0.719	0.950 (0.700 to 1.280)
GEJ II	266	140	0.609	1.080 (0.790 to 1.480)
GEJ III	76	17	0.365	1.550 (0.600 to 3.990)
cT-Stage				
I/II	65	110	0.228	1.450 (0.790 to 2.660)
III	549	166	0.010*	0.730 (0.570 to 0.930)
IV	62	9	0.822	0.890 (0.310 to 2.560)
cN-Stage				
cN-	100	176	0.422	0.830 (0.530 to 1.300)
cN+	600	110	0.147	0.810 (0.610 to 1.080)
Tumor Grade				
I/II	293	136	0.086	1.350 (0.960 to 1.900)
III/IV	329	151	0.088	0.790 (0.600 to 1.040)

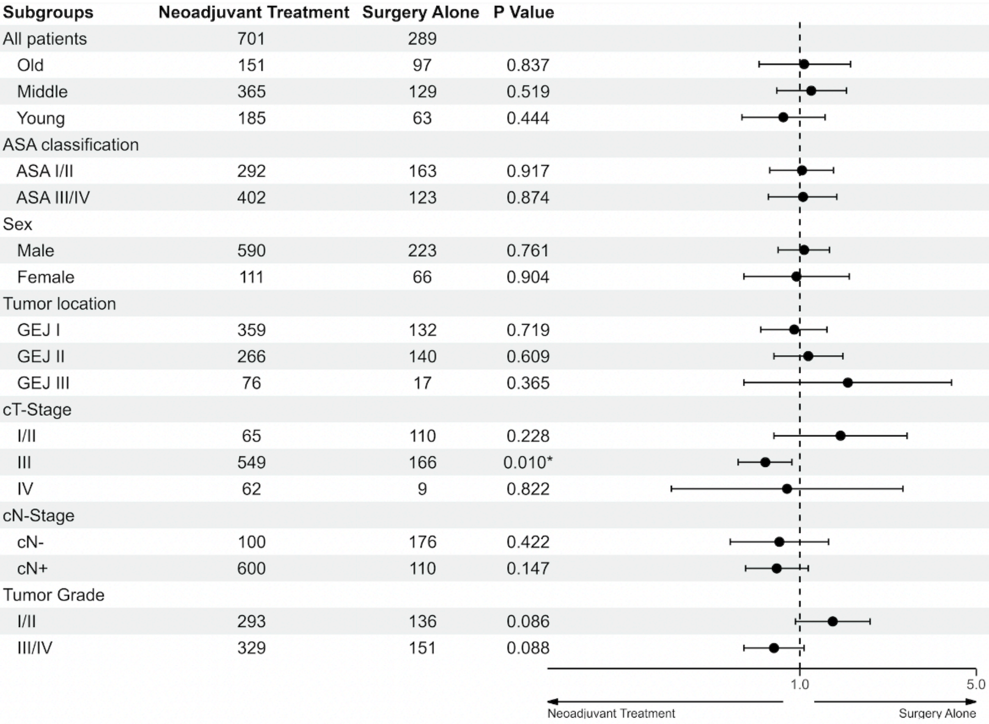

Figure 5: Multivariate Disease-Free Survival Forest Plot

Subgroups	Neoadjuvant Treatment	Surgery Alone	P Value	Hazard Ratio (95% CI)
All patients	701	289		
Old	151	97	0.110	0.680 (0.430 to 1.090)
Middle	365	129	0.061	0.710 (0.500 to 1.020)
Young	185	63	0.081	0.690 (0.450 to 1.050)
ASA classification				
ASA I/II	292	163	0.053	0.720 (0.520 to 1.000)
ASA III/IV	402	123	0.025*	0.670 (0.480 to 0.950)
Sex				
Male	590	223	0.010*	0.700 (0.530 to 0.920)
Female	111	66	0.178	0.700 (0.420 to 1.170)
Tumor location				
GEJ I	359	132	0.007*	0.620 (0.440 to 0.880)
GEJ II	266	140	0.126	0.760 (0.540 to 1.080)
GEJ III	76	17	0.747	0.850 (0.320 to 2.260)
cT-Stage				
I/II	65	110	0.843	1.070 (0.530 to 2.160)
III	549	166	0.003*	0.670 (0.510 to 0.870)
IV	62	9	0.567	0.730 (0.240 to 2.160)
cN-Stage				
cN-	100	176	0.041*	0.620 (0.390 to 0.980)
cN+	600	110	0.050	0.740 (0.540 to 1.000)
Tumor Grade				
I/II	293	136	0.522	0.880 (0.610 to 1.290)
III/IV	329	151	0.001*	0.590 (0.430 to 0.810)

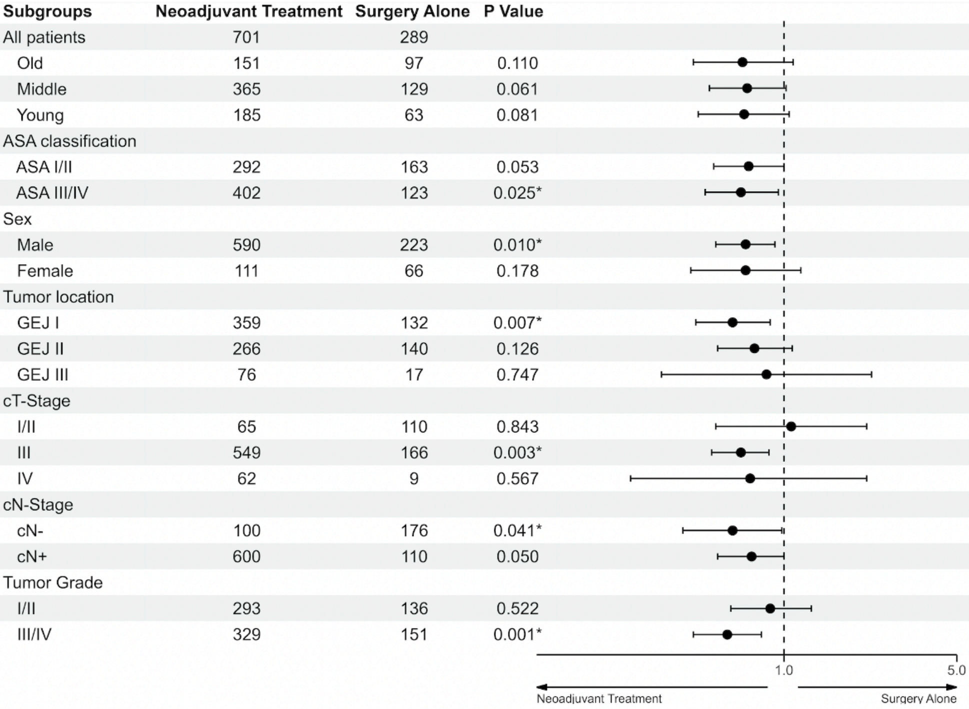

Figure 6: FLOT-Subanalysis OS-univariate

Subgroups	Neoadjuvant Treatment	Surgery Alone	P Value	Hazard Ratio (95% CI)
All patients	381	289		
Old	79	97	0.985	0.996 (0.649 to 1.528)
Middle	197	129	0.378	0.858 (0.610 to 1.206)
Young	105	63	<0.001*	0.409 (0.248 to 0.675)
ASA classification				
ASA I/II	156	163	0.013*	0.643 (0.454 to 0.910)
ASA III/IV	219	123	0.068	0.737 (0.531 to 1.023)
Sex				
Male	314	223	0.002*	0.661 (0.507 to 0.861)
Female	67	66	0.519	0.847 (0.512 to 1.402)
Tumor location				
GEJ I	167	132	0.001*	0.547 (0.383 to 0.779)
GEJ II	169	140	0.208	0.799 (0.563 to 1.133)
GEJ III	45	17	0.829	0.917 (0.417 to 2.015)
cT-Stage				
I/II	36	110	0.654	0.840 (0.393 to 1.797)
III	288	166	<0.001*	0.538 (0.412 to 0.703)
IV	36	9	0.142	0.494 (0.193 to 1.265)
cN-Stage				
cN-	40	176	0.181	0.610 (0.295 to 1.258)
cN+	341	110	<0.001*	0.545 (0.406 to 0.731)
Tumor Grade				
I/II	170	136	0.503	0.876 (0.594 to 1.291)
III/IV	158	151	<0.001*	0.556 (0.406 to 0.762)

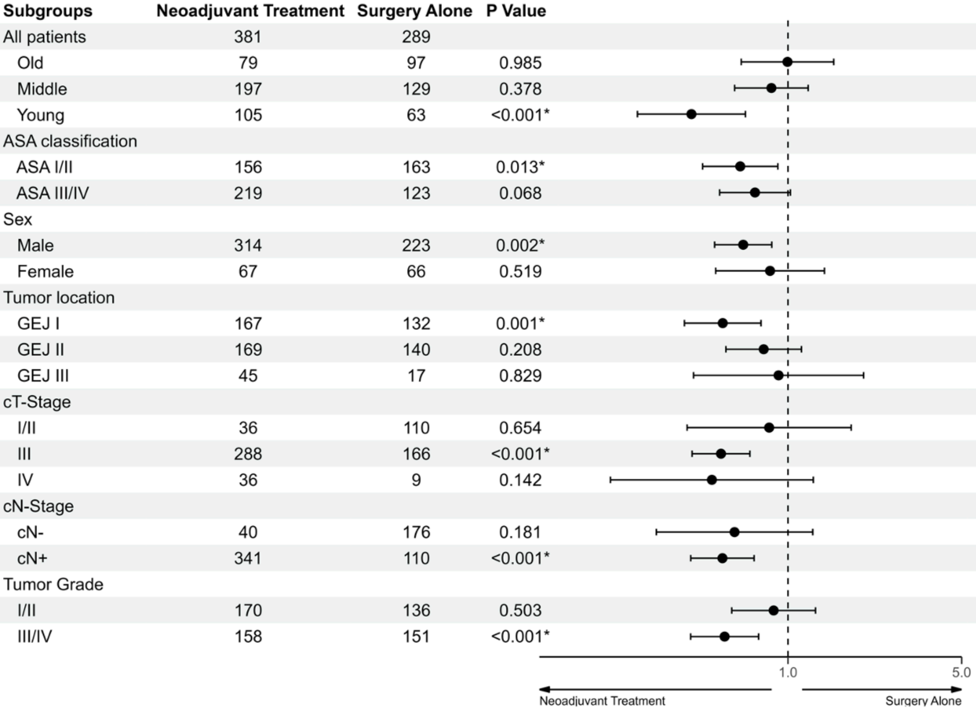

Figure 7: FLOT-Subanalysis OS-multivariate

Subgroups	Neoadjuvant Treatment	Surgery Alone	P Value	Hazard Ratio (95% CI)
All patients	381	289		
Old	79	97	0.059	0.618 (0.375 to 1.019)
Middle	197	129	0.015*	0.617 (0.418 to 0.910)
Young	105	63	<0.001*	0.352 (0.200 to 0.619)
ASA classification				
ASA I/II	156	163	0.003	0.551 (0.370 to 0.822)
ASA III/IV	219	123	0.002	0.538 (0.367 to 0.790)
Sex				
Male	314	223	<0.001*	0.511 (0.370 to 0.705)
Female	67	66	0.228*	0.707 (0.403 to 1.242)
Tumor location				
GEJ I	167	132	<0.001*	0.494 (0.327 to 0.747)
GEJ II	169	140	0.021*	0.621 (0.415 to 0.931)
GEJ III	45	17	0.071*	0.462 (0.200 to 1.067)
cT-Stage				
I/II	36	110	0.177	0.435 (0.130 to 1.458)
III	288	166	<0.001*	0.570 (0.418 to 0.776)
IV	36	9	0.033*	0.331 (0.120 to 0.913)
cN-Stage				
cN-	40	176	0.097	0.533 (0.254 to 1.120)
cN+	341	110	<0.001*	0.546 (0.396 to 0.755)
Tumor Grade				
I/II	170	136	0.097	0.697 (0.454 to 1.068)
III/IV	158	151	<0.001*	0.464 (0.323 to 0.667)

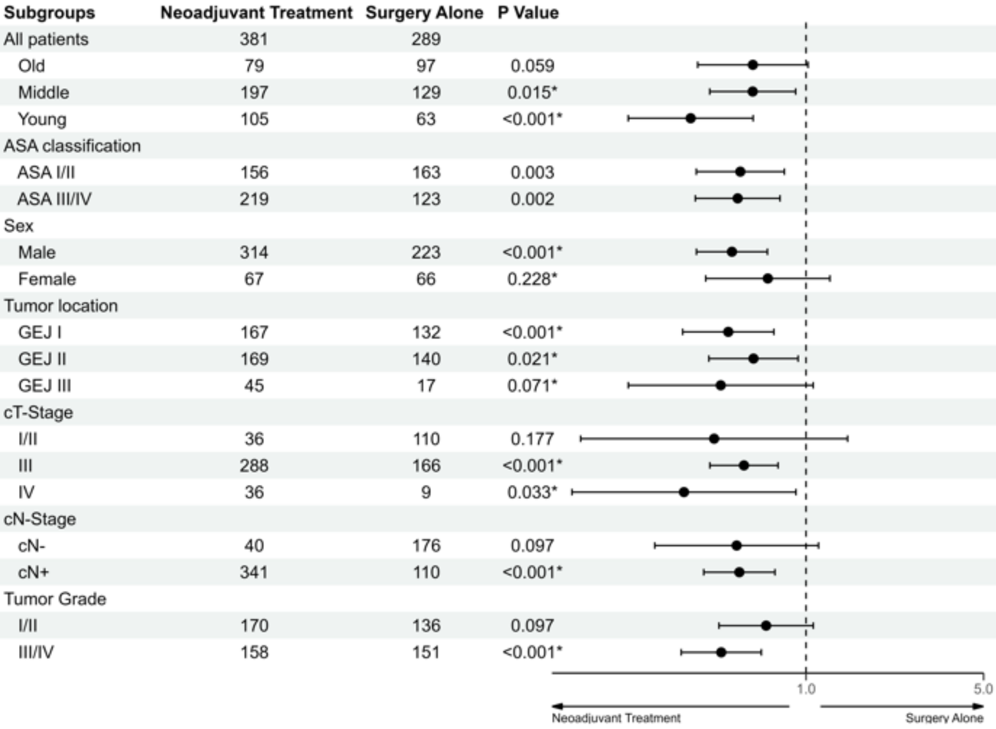

Figure 8: FLOT-Subanalysis DFS-univariate

Subgroups	Neoadjuvant Treatment	Surgery Alone	P Value	Hazard Ratio (95% CI)
All patients	381	289		
Old	79	97	0.365	1.272 (0.756 to 2.140)
Middle	197	129	0.201	1.265 (0.882 to 1.812)
Young	105	63	0.209	0.756 (0.489 to 1.169)
ASA classification				
ASA I/II	156	163	0.965	1.008 (0.707 to 1.436)
ASA III/IV	219	123	0.561	1.109 (0.783 to 1.571)
Sex				
Male	314	223	0.762	1.043 (0.794 to 1.370)
Female	67	66	0.348	1.293 (0.756 to 2.211)
Tumor location				
GEJ I	167	132	0.798	0.954 (0.665 to 1.368)
GEJ II	169	140	0.281	1.214 (0.853 to 1.726)
GEJ III	45	17	0.443	1.486 (0.540 to 4.091)
cT-Stage				
I/II	36	110	0.087	1.886 (0.912 to 3.902)
III	288	166	0.050*	0.758 (0.574 to 0.999)
IV	36	9	0.677	0.784 (0.249 to 2.465)
cN-Stage				
cN-	40	176	0.920	1.035 (0.532 to 2.015)
cN+	341	110	0.208	0.819 (0.599 to 1.118)
Tumor Grade				
I/II	170	136	0.088	1.402 (0.951 to 2.068)
III/IV	158	151	0.413	0.873 (0.631 to 1.209)

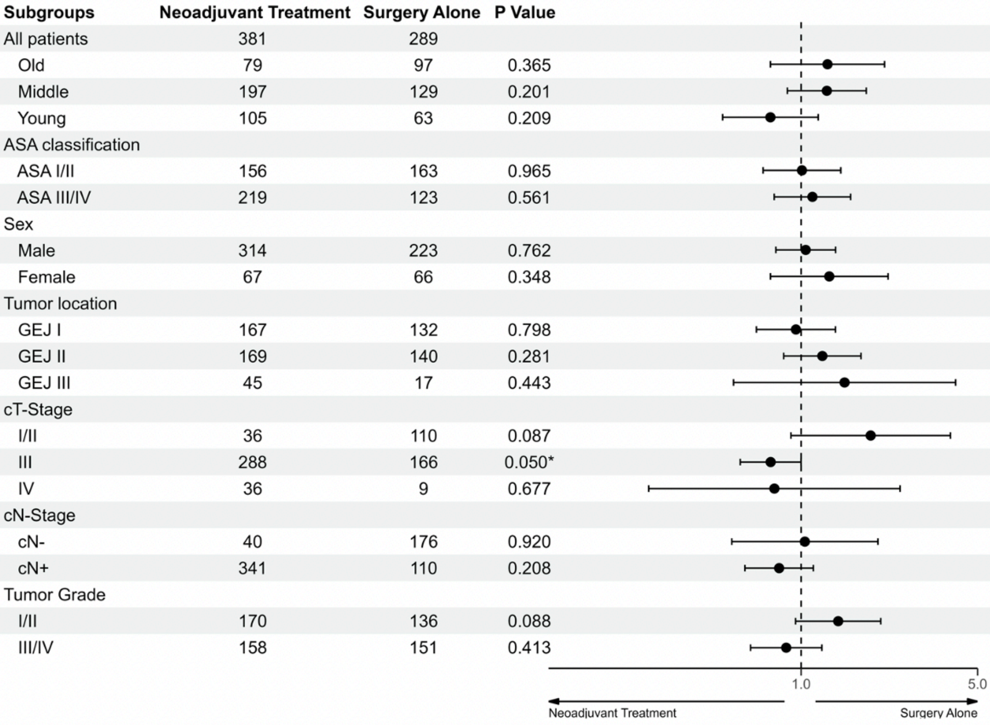

Figure 9: FLOT-Subanalysis DFS-multivariate

Subgroups	Neoadjuvant Treatment	Surgery Alone	P Value	Hazard Ratio (95% CI)
All patients	381	289		
Old	79	97	0.516	0.819 (0.448 to 1.496)
Middle	197	129	0.495	0.868 (0.577 to 1.305)
Young	105	63	0.064	0.623 (0.378 to 1.029)
ASA classification				
ASA I/II	156	163	0.287	0.800 (0.530 to 1.206)
ASA III/IV	219	123	0.165	0.754 (0.506 to 1.124)
Sex				
Male	314	223	0.050*	0.718 (0.515 to 1.001)
Female	67	66	0.819	1.071 (0.594 to 1.933)
Tumor location				
GEJ I	167	132	0.055	0.660 (0.431 to 1.010)
GEJ II	169	140	0.616	0.901 (0.600 to 1.353)
GEJ III	45	17	0.613	0.759 (0.261 to 2.205)
cT-Stage				
I/II	36	110	0.516	1.343 (0.551 to 3.275)
III	288	166	0.092	0.759 (0.551 to 1.045)
IV	36	9	0.345	0.555 (0.164 to 1.883)
cN-Stage				
cN-	40	176	0.533	0.803 (0.404 to 1.598)
cN+	341	110	0.131	0.769 (0.547 to 1.081)
Tumor Grade				
I/II	170	136	0.763	0.936 (0.607 to 1.442)
III/IV	158	151	0.044*	0.675 (0.461 to 0.989)

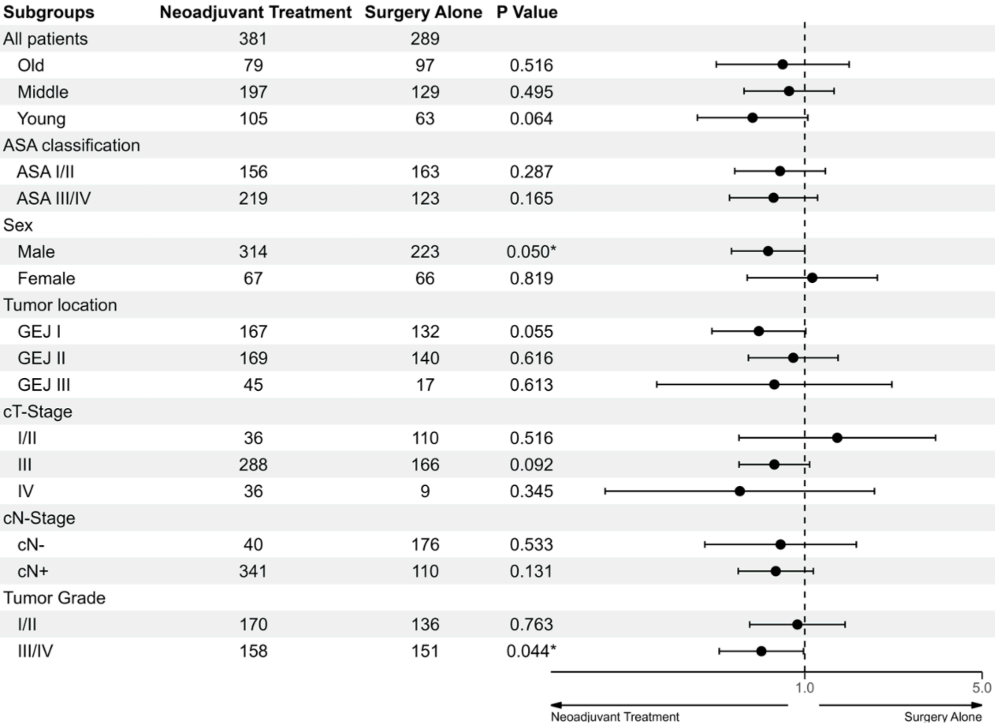

Table 1: Other Values Age comparisons

	Young			Old			
	**Neoadjuvant Treatment**	**Surgery Alone**	**P Value**	**Neoadjuvant Treatment**	**Surgery Alone**	**P Value**	**Young vs. Old**
**Stage**			<0.001			<0.001	0.043
**IIa**	3 (1.66%)	0 (0%)		0 (0%)	1 (1.07%)		
**IIb**	0 (0%)	16 (26.67%)		3 (2.04%)	31 (33.33%)		
**III**	174 (96.13%)	43 (71.67%)		143 (97.28%)	59 (63.44%)		
**Iva**	4 (2.21%)	1 (1.67%)		1 (0.68%)	2 (2.15%)		
**cTx or cN+**	8	4		5	6		
**Causes of Death**			0.695			0.687	<0.001
**Cancer-related**	37 (88.10%)	30 (90.91%)		30 (61.22%)	30 (65.22%)		
**related to postoperative complications**	3 (7.14%)	1 (3.03%)		12 (24.49%)	9 (19.57%)		
**other reasons**	2 (4.76%)	2 (6.06%)		7 (14.29%)	7 (15.22%)		
**not available**	24	5		25	14		
**Type of neoadjuvant therapy**							0.121
**RCT**	29 (15.68%)			37 (24.50%)			
**FLOT**	105 (56.76%)			79 (52.32%)			
**CT other**	51 (27.57%)			35 (23.18%)			
**EOX/ECX**	37			24			
**ECF/EOF**	7			6			
**PLF**	4			1			
**TCF/Gastrotax**	3			2			
**FOLFOX**	0			2			

Table 2: Neoadjuvant Young vs. Old and Upfront Surgery Young vs. Old

	Young Neoadjuvant	Old Neoadjuvant	P Value	Young Surgery Alone	Old Surgery Alone	P Value
**Total N**	185	151		63	97	
**Age (years)**			<0.001			<0.001
**median (SD)**	52.4 (6.27)	74.64 (3.04)		49.94 (5.78)	76.78 (4.52)	
**ASA**			<0.001			0.059
**1/2**	97 (53.30%)	50 (33.33%)		43 (68.25%)	47 (38.45%)	
**3/4**	85 (46.70%)	100 (66.67%)		20 (31.75%)	50 (51.55%)	
**missing**	3	1		0	0	
**Sex**			0.286			0.572
**Female**	33 (17.84%)	34 (22.52%)		15 (23.81%)	27 (27.84%)	
**Male**	152 (82.16%)	117 (77.48%)		48 (76.19%)	70 (72.16%)	
**Tumor-Location**			0.031			0.518
**GEJ 1/mE**	87 (47.03%)	73 (48.34%)		24 (38.10%)	42 (43.30%)	
**GEJ 2**	81 (43.78%)	51 (33.77%)		33 (55.38%)	50 (51.55%)	
**GEJ 3**	17 (9.19)	27 (17.88%)		6 (9.52%)	5 (5.15%)	
**cT-Stage**			0.409			0.251
**cT1**	3 (1.66%)	0 (0%)		0 (0%)	1 (1.05%)	
**cT2**	11 (6.08%)	10 (6.80%)		19 (31.15%)	39 (41.05%)	
**cT3**	146 (80.66%)	123 (83.67%)		39 (63.93%)	54 (56.84%)	
**cT4**	21 (11.60%)	14 (9.52%)		3 (4.92%)	1 (1.05%)	
**cTx**	4	4		2	2	
**cN-Stage**			0.857			0.156
**cN-**	22 (11.89%)	17 (11.26%)		41 (66.13%)	52 (54.74%)	
**cN+**	163 (88.11%)	134 (88.74%)		21 (33.87%)	43 (45.26%)	
**cNx**	0	0		1	2	
**Tumor Grade**			0.422			0.984
**G1**	7 (4.32%)	2 (1.53%)		3 (4.76%)	4 (4.17%)	
**G2**	68 (41.98%)	59 (45.04%)		21 (33.33%)	32 (33.33%)	
**G3 + 4**	87 (53.70%)	70 (53.44%)		39 (61.90%)	60 (62.50%)	
**missing**	22	20		0	1	
**Type of neoadjuvant therapy**			0.121			
**FLOT**	105 (56.76%)	79 (52.32%)				
**CT other**	51 (27.57%)	35 (23.18%)				
**RCT**	29 (15.68%)	37 (24.50%)				
**Type of Surgery**			0.151			0.453
**ILE**	137 (74.05%)	101 (66.89%)		35 (55.56%)	48 (49.48%)	
**THG**	48 (25.95%)	50 (33.11%)		28 (44.44%)	49 (50.52%)	

Table 3: Neoadjuvant Treatment Received subanalyses

Characteristic	All patients			FLOT			Other chemotherapy			Radiochemotherapy		
	Young NAT, N = 185[1]	Old NAT, N = 151[1]	p-value[2]	Young NAT, N = 105[1]	Old NAT, N = 79[1]	p-value[2]	Young NAT, N = 51[1]	Old NAT, N = 35[1]	p-value[2]	Young NAT, N = 29[1]	Old NAT, N = 37[1]	p-value[3]
**Discontinuation of neoadjuvant treatment**	11 (5.9%)	21 (14%)	**0.013**	4 (3.8%)	10 (13%)	**0.023**	6 (12%)	9 (26%)	0.094	1 (3.4%)	2 (5.4%)	>0.999
**Reason for Discontinuation**			**0.020**			0.505			0.119			0.333
Non-Compliance	0 (0%)	1 (4.8%)					0 (0%)	1 (11%)				
Non-Response	7 (64%)	4 (19%)		1 (25%)	1 (10%)		5 (83%)	3 (33%)		1 (100%)	0 (0%)	
Toxicity	4 (36%)	16 (76%)		3 (75%)	9 (90%)		1 (17%)	5 (56%)		0 (0%)	2 (100%)	
**Dose reduction**	23 (12%)	61 (40%)	**<0.001**	13 (12%)	45 (57%)	**<0.001**	9 (18%)	14 (40%)	**0.021**	1 (3.4%)	2 (5.4%)	>0.999
**Reason for dose reduction**			**0.013**			**0.019**				0 (NA%)	0 (NA%)	
Reduced tolerability	2 (17%)	23 (58%)		2 (22%)	23 (66%)							
Severe side effects	10 (83%)	17 (43%)		7 (78%)	12 (34%)		3 (100%)	5 (100%)				

[1] n (%)

[2] Pearson's Chi-squared test; Fisher's exact test

[3] Fisher's exact test

Table 5: Relative Dose intensity

**FLOT**	**Young** N=105	**Old** N=95	**p-value**[1]
Combined RDI	0.976 (0.074)	0.844 (0.163)	**<0.001**
5-Fluorouracil	0.989 (0.049)	0.956 (0.123)	**0.026**
Oxaliplatin	0.992 (0.043)	0.965 (0.107)	**0.036**
Docetaxel	0.946 (0.185)	0.613 (0.440)	**<0.001**
Number of Patients with RDI ≤0.85	9 (8.6%)	38 (40.0%)	**<0.001**
Number of Patients with RDI ≤0.80	6 (5.7%)	36 (37.9%)	**<0.001**

Mean (SD), [1]calculated by T-Test (Welch, Two Sample); Pearson's Chi-squared test, RDI=Relative Dose Intensity

Table 6: Traditional Age cut-offs Early-onset (<50) and Late-onset (>75)

Subgroups	N	Median OS [months]	p	Median DFS [months]	p
Early-Onset Neoadjuvant	81	Not reached (95%CI:43.2 - not reached)	0.031	76.5 (95%CI:37.3-not reached)	0.430
Early-Onset Upfront	33	34.0 (95%CI:21.5 - not reached)		23.7 (95%CI:26.0-not reached)	
Late-Onset Neoadjuvant	70	23.9 (95%CI:13.8 - not reached)	0.881	24.3 (95%CI:15.7-36.7)	0.684
Late-Onset Upfront	60	51.9 (95%CI:17.5 - not reached)		24.7 (95%CI:14.0-46.1)	

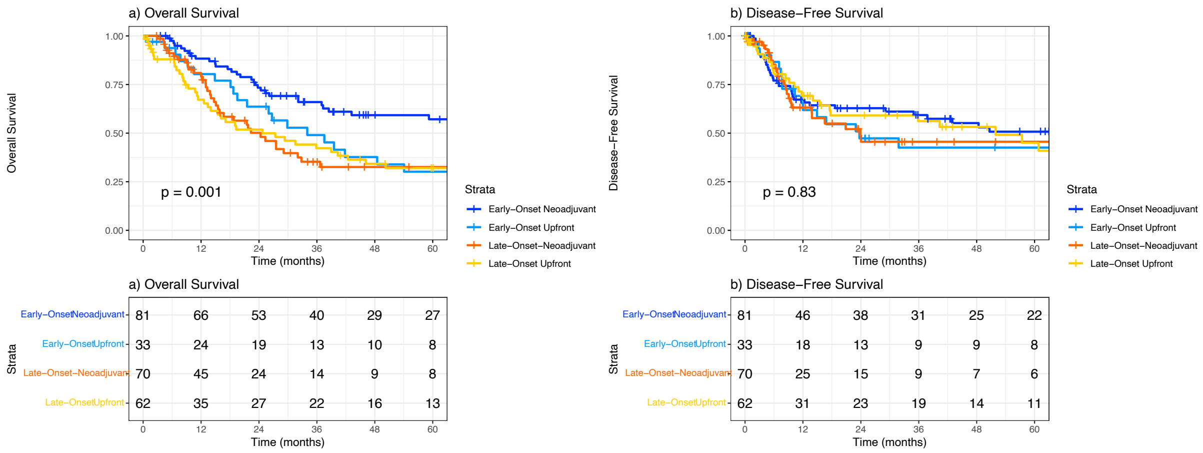

Supplement: SUPPLEMENTARY MATERIAL [file js9-109-3804-s001.pdf]
